# Plasma-Activated Acidic Electrolyzed Water: A New Food Disinfectant for Bacterial Suspension and Biofilm

**DOI:** 10.3390/foods11203241

**Published:** 2022-10-17

**Authors:** Yaping Heng, Ming Wang, Hongwei Jiang, Shumin Gao, Jin Zhang, Jinlin Wan, Tingji Song, Zhandong Ren, Yuchan Zhu

**Affiliations:** School of Chemical and Environmental Engineering, Wuhan Polytechnic University, Wuhan 430023, China

**Keywords:** food processing, plasma-activated acidic electrolyzed water, *B. subtilis*, sterilization effect

## Abstract

Food-borne diseases are widespread all over the world, and food safety has attracted much attention. This study is the first to use plasma to activate acidic electrolyzed water (AEW) to obtain a new disinfectant for food processing. The germicidal efficacy of plasma-activated acidic electrolyzed water (PA-AEW) on *B. subtilis* suspension and biofilm was investigated. Furthermore, the synergistic effect of different bactericidal factors was inferred by investigating the physicochemical parameters of PA-AEW and the influencing factors of bactericidal effect. The results demonstrate that PA-AEW is a highly effective and rapid disinfectant. The killing logarithm (KL) value of PA-AEW on *B. subtilis* suspension could reach 2.33 log_10_CFU/mL with a sterilization time of 10 s, which is significantly higher than that of AEW (KL = 0.58 log_10_CFU/mL) and plasma-activated water (PAW) (KL = 0.98 log_10_CFU/mL) (significant difference, *p* < 0.01). Moreover, the KL value of the *B. subtilis* biofilm of PA-AEW was 2.41 log_10_CFU/mL, better than that of PAW and AEW (significant difference, *p* < 0.01), indicating that PA-AEW has important application prospects in food processing. The synergistic effect should come from the interaction between reactive chlorine species (RCS) and reactive oxygen and nitrogen species (RONS) in PA-AEW.

## 1. Introduction

Food-borne diseases are widespread all over the world, and the related food safety in the process of food production and processing has also attracted extensive attention. Disinfection is the key step to control food-borne pathogenic microorganisms. Therefore, new environmentally friendly sterilization technology has been widely concerned in the food industry, including ozone, plasma-activated water (PAW), acidic electrolyzed water (AEW), ultrasound technology (US), pulsed electric field, etc. [1,2,3].

AEW has the characteristics of high sterilization efficiency and a wide sterilization spectrum. The main bactericidal factor in AEW is reactive chlorine species (RCS), mainly in the form of HClO. The bactericidal efficiency of HClO is 80 times that of ClO^−^ [4,5]. AEW can sterilize a variety of microorganisms [6,7,8,9,10], and is widely used in food sterilization, including vegetables, fruits, meat, fish, eggs and other types of foods [11,12,13,14,15,16,17,18]. Among them, the Bacillus species is responsible for food spoilage and food-borne disease. In Australia, the United Kingdom and South Korea, the microbial limit standards in ready-to-eat food should not exceed 10,000 CFU/g, 100,000 CFU/g and 1000 CFU/g, respectively. However, the sterilization efficiency of AEW for Bacillus species is not high [19,20,21,22,23,24], requiring a long sterilization time (>2 min) or a high RCS (>60 mg/L) to achieve a high sterilization efficiency. When AEW was used alone for sterilization, the RCS content of 60 mg/L and treatment for 5 min could reduce *B. cereus* by 2.11 log CFU/cm^2^ [21]. However, for the inactivation of *B. subtilis* spores with 6 log CFU/cm^2^, the RCS of AEW was 120 mg/L and the treatment time was 2 min [19]. Even if the combination of AEW and other methods is used for sterilization, a long treatment time is also required. For example, the combination of AEW and US treatment required 3 min to reduce *B. cereus* by about 3.0 log CFU/g [23]. The combination of AEW (RCS = 80 mg/L) with mild heat and US treatment for 10 min could result in a reduction of 4.0 log CFU/cm^2^ for *Bacillus cereus* biofilms [22].

The cold atmospheric pressure plasma (CAPP) is nonequilibrium plasma. When the CAPP comes into contact with water, some reactive oxygen and nitrogen species (RONS) will be produced. The RONS include hydrogen peroxide (H_2_O_2_), ozone (O_3_), nitrate (NO_3_^−^), nitrite (NO_2_^−^), hydroxyl radicals (•OH), singlet oxygen (^1^O_2_), superoxide (O_2_^−^), nitric oxide (•NO), peroxynitrite (ONOO^−^) and so on [25,26,27]. The RONS have the characteristics of sterilization efficiency and environmental friendliness. Therefore, plasma activation technology can be used to prepare a new disinfectant, called plasma-activated water (PAW). PAW has been used to inactivate many food-borne pathogenic microorganisms [28,29,30,31,32,33,34,35,36,37,38,39,40,41,42]. However, disinfection with PAW alone requires a long sterilization time, which may be a few minutes or more. In the investigations of Frías et al. [30] and Han et al. [29], the PAW treatment times of *S. Enteritidis* or *E. coli* O157:H7 and *S. Typhimurium* or *E. coli* O157:H7 were 15 and 40 min. For jerky [38] and grape [39] samples, the PAW treatment time was 30 min. In Royintarat’s research [34], for chicken samples with a thickness of 4 mm, the PAW treatment time was as long as 60 min. Furthermore, for *B. cereus* spores that can resist harsh environments, PAW treatment for at least 60 min was needed in Liao and Bai’s research [31,40]. As for biofilm, the PAW treatment time was 30 min in Xu’s report [32]. In addition, PAW treatment will not affect the biochemical or sensory characteristics of food. For fruits, such as grapes [39], PAW treatment did not significantly change their total soluble solids, reducing sugar, pH value, titratable acidity, hardness, surface color, total phenols, vitamin C and antioxidant properties. For apples [37], PAW treatment did not brown their surface, but did not affect their hardness or titratable acidity. For bean products [30], it promoted the control of pathogenic microorganisms on tofu, while maintaining its physical and functional characteristics. In addition, Liao et al. [31] and Han et al. [29] showed that PAW treatment did not adversely affect the texture and sensory quality of cooked rice, and did not affect the color, texture or pH value of Korean rice cakes.

In order to further improve the germicidal efficacy of AEW and PAW, for the first time, we used plasma to activate AEW in this study. The germicidal efficacy of plasma-activated AEW (PA-AEW) against *Bacillus subtilis* suspension and biofilm was investigated. The plasma activation effect of AEW with different RCS content was also studied, and the relationship between the bactericidal efficiency of PA-AEW and the HClO content in AEW was preliminarily analyzed. Furthermore, the effects of plasma activation time on the physicochemical parameters and long-lived RONS of PA-AEW were also investigated. According to the RONS content in PA-AEW tested above, the modified AEW was prepared, and the active bactericidal factors in PA-AEW were preliminarily inferred by comparing the bactericidal effects of PA-AEW and M-AEW. Finally, to further verify the feasibility of application of PA-AEW in food processing, the bactericidal effect of PA-AEW on bacterial biofilm was also investigated.

## 2. Materials and Methods

### 2.1. AEW Preparation

The 0.1 wt % NaCl was electrolyzed to produce the AEW in an anion-exchange membrane electrolytic cell with a volume of 500 mL. The anode was the IrO_2_-Ta_2_O_5_-TiO_2_ composite oxide and the cathode was the Ti plate. The electrode area was 75 cm^2^. The current density was fixed at 3 mA cm^−2^ and the electrolysis time was 50 min.

### 2.2. PAW and PA-AEW Preparation

**Scheme 1:**
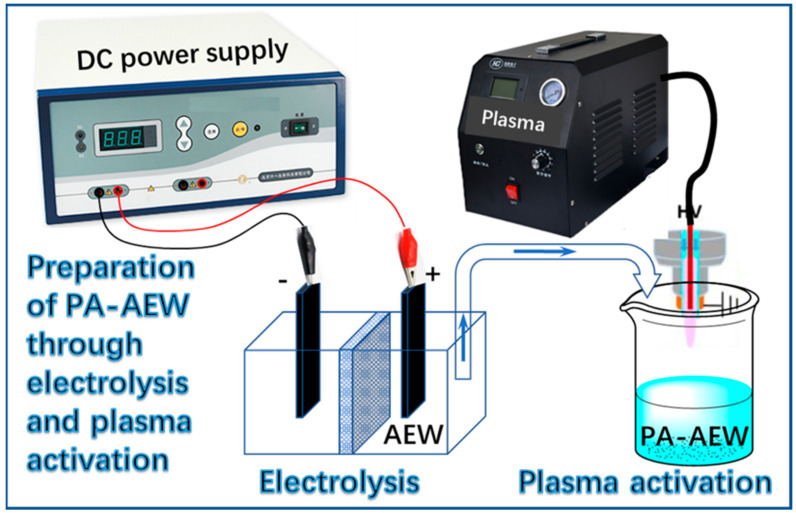
Schematic illustration of the preparation of PA-AEW.

The preparation of PA-AEW is shown in Scheme 1. An atmospheric plasma jet with air as the excitation gas source (Hongcheng Electronic Technology Co., Ltd., Shenzhen, China) was used in this study. During the preparation of PAW, the plasma jet was placed 80 mm above the surface of AEW. The volume of AEW was 200 mL. The compressed air pressure was about 0.16 MPa with a flow rate of 30 L/min. The input voltage and current were 220 V and 3 A, respectively. The plasma activation time was 3~15 min. The preparation process of PAW was the same as that of PA-AEW, except sterile water was used instead of AEW.

### 2.3. pH, Oxidation-Reduction Potential (ORP) and Electrical Conductivity (EC) Analysis

The values of pH and ORP were analyzed by a pH meter (3-Star, Thermo Orion, Waltham, MA, USA) bearing pH and ORP electrodes. The EC value was analyzed by a conductivity meter with a conductivity electrode (DDS-307, INESA Scientific Instrument Co., Ltd., Shanghai, China).

### 2.4. H_2_O_2_, NO_3_^−^ and NO_2_^−^ Analysis

The concentrations of H_2_O_2_, NO_3_^−^ and NO_2_^−^ were determined by spectrophotometry. Potassium permanganate (KMnO_4_) underwent the redox reaction with H_2_O_2_, so the concentration of H_2_O_2_ can be determined at 525 nm by spectrophotometry. NO_3_^−^ contains a -N=O group, which has a strong absorption peak at 220 nm, so the concentration of NO_3_^−^ was determined by spectrophotometry. NO_2_^−^ can react with p-aminobenzenesulfonic acid (H_2_N-C_6_H_5_-SO_3_H, as a diazotization reagent) in a weak acid environment, and then react with naphthylethylenediamine hydrochloride (C_12_H_14_N_2_·2HCl, coupling reagent) to generate purple dye. Therefore, the concentration of NO_2_^−^ was determined at the absorbance of 540 nm.

### 2.5. RCS and HClO Analysis

The concentration of total RCS dissolved in the solution was determined using the TMB (3,3′,5,5′-tetramethylbenzidine) colorimetric method. In this method, the TMB was oxidized to form a yellow product and its concentration was analyzed immediately using a spectrophotometer (TU-1900, Beijing Purkinje General Instrument Co., Ltd., Beijing, China) at 449 nm. The content of HClO was measured directly by a spectrophotometer at 233 nm, where the absorption coefficient ε is 100 [24]. These were measured three times and the average values were obtained.

### 2.6. Bacterial and Culture

*B. subtilis* (ATCC6633, purchased from Shanghai Luwei Tech. Co., Ltd., Shanghai, China) and *E. coli* (ATCC8739, purchased from Guangdong Huankai Microbial Sci. & Tech. Co., Ltd., Guangdong, China) were used as representatives of Gram-positive and Gram-negative bacteria, respectively. The stock cultures were transferred into nutrient broth (NB) and incubated at 37 °C for 24 h. Following incubation, 10 mL of culture was sedimented by centrifugation (3000× *g* for 10 min at 4 °C), washed and resuspended by 5 mL of 0.85% sterile saline solution to obtain a final cell concentration of 10^7^~10^9^ CFU/mL. The bacterial population was confirmed by plating 0.1 mL portions of appropriately diluted cultures on tryptone soya agar (TSA) plates and incubating at 37 °C for 24 h.

### 2.7. Sterilization Effect of Bacterial Suspension

For each replicate, a 1 mL aliquot of prepared inoculum (10^7^~10^9^ CFU/mL) was added to a sterile test tube. Next, 9 mL of PA-AEW (AEW, PAW) was added to the tube containing 1 mL of the prepared inoculums for 10 s. Then, a 1 mL sample was transferred to a tube containing 9 mL of neutralizer (0.85 wt % NaCl + 0.5 wt % sodium thiosulphate) to stop sterilization. After neutralization, the treated and control (untreated) samples were then serially diluted in sterile 0.85 wt % saline solution dilution blanks. Following serial dilution, 1 mL of each sample was plated on TSA plates and incubated at 37 °C for 24 h. The survival populations of *B. subtilis* and *E. coli* were determined by the colony counting using plate count method.

### 2.8. Sterilization Effect of Bacillus subtilis Biofilm

The biofilms were grown on 304 stainless steel sheets (1 × 1 cm^2^). The pretreatment process of a stainless steel sheet includes ethanol cleaning to remove surface grease, 5 mol/L hydrochloric acid ultrasonic cleaning to remove inorganic impurities and ultrapure water washing 3–5 times. The treated stainless steel sheet was placed in a test tube containing 10 mL nutrient broth culture medium, then autoclaved at 121 °C for 30 min. We added 0.1 mL of *B. subtilis* suspension (10^8^ CFU/mL) into the test tube, and then inoculated at 37 °C and 150 r/min for bacterial attachment. The biofilm growth needs to be carried out continuously for 7 days, and the bacterial culture solution must be changed every 24 h. The stainless steel sheet with biofilms cultured for 7 days were washed gently with PBS to remove any loose bacteria. Then, the stainless steel sheet was put into 10 mL PA-AEW for sterilization for 10 s, and then quickly transferred into 10 mL of neutralizer to terminate sterilization for 10 min. The stainless steel sheet was removed and put into 10 mL of normal saline and sonicated for 15 min. Then, the solution was diluted to the appropriate concentration and incubated at 37 °C for 24 h.

### 2.9. Visualization of the Cell Using Fluorescence and Scanning Electron Microscopy

Scanning electron microscope was used to evaluate cell morphology after PA-AEW treatment. Bacterial cells treated with the PA-AEW treatment were fixed with 2.5% glutaraldehyde overnight and then washed three times with PBS. Then, the cells were dehydrated in an ascending ethanol gradient (30%, 50%, 70%, 80%, 90%, 95% and 100% twice for 15 min each), and then the ethanol was displaced by isoamyl acetate until desiccation. The samples were gilded and observed with a SU8100 field-emission SEM (Hitachi, Japan).

The cell viability can be evaluated with a fluorescence probe (propidium iodide, PI) through fluorescence microscopy. The red fluorescing PI can only enter cells with injured cytoplasmic membranes, where it binds to nucleic acids and produces red fluorescence. Fluorescence microscopy (FL) analysis was conducted on a BX51 (Olympus, Tokyo, Japan).

### 2.10. Statistical Analysis

All experiments were performed in duplicates with three replicates of each analysis and treatment. The microbial counts are expressed as log_10_CFU/mL of the sample. Furthermore, the killing logarithm value (log_10_CFU/mL reduction) on bacterial population was computed. The values reported for plate counts were the mean ± standard deviation values of individual samples with duplicate plates for each sample. Data were subjected to one-way analysis of variance (ANOVA) using Microsoft Excel 2016. The differences between these means were determined by Tukey’s multiple comparisons with significance set at *p* < 0.05.

## 3. Results and Discussion

**Figure 1:**
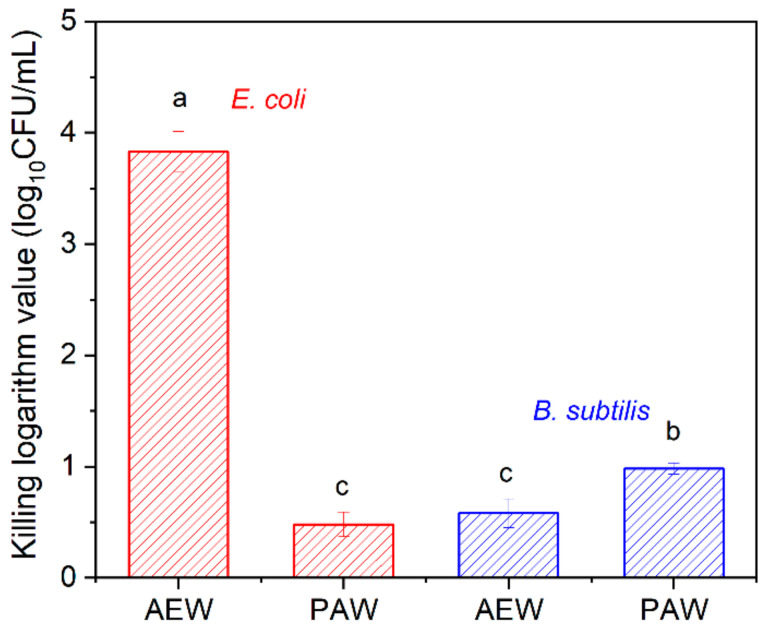
Sterilization effects of PAW and AEW on the bacterial suspension of *E. coli* and *B. subtilis*. Values with different lowercase letters above graph bars represent a significant difference (*p* < 0.05).

At present, PAW and AEW are new disinfectants that have received extensive attention. Both disinfectants have the characteristics of high efficiency, broad spectrum and environmental friendliness. Firstly, taking *E. coli* and *B. subtilis* as representatives of Gram-negative bacteria and Gram-positive bacteria, the sterilization effects of the two disinfectants are compared in Figure 1. In order to highlight the high efficiency of the two disinfectants, a short sterilization time was set at 10 s. For the AEW, it had a good sterilization effect on *E. coli*, and its killing logarithm (KL) value can reach 3.83 log_10_CFU/mL. However, for *B. subtilis*, the sterilization time of 10 s was not enough, and its KL value was only 0.58 log_10_CFU/mL. There was a significant difference between them (*p* < 0.01). This is consistent with the results reported in previous literature [19,21], in which the KL values of *B. subtilis* spores and *Bacillus cereus* are 0.47 and 1.53 log_10_CFU/mL with the killing times of 2 and 1 min. For PAW, its sterilization effect was not as good as AEW. The KL values of PAW to *E. coli* and *B. subtilis* were only 0.48 and 0.98 log_10_CFU/mL, respectively. This is because the treatment of 10 s is too short in PAW sterilization. In the investigation of Frías et al. [30], the PAW treatment time was 15 min, and the KL values of *E. coli* O157:H7 were 0.2~0.6 log_10_CFU/mL, which were close to the germicidal efficiency in this study. In Han’s study [29], the PAW treatment was increased to 40 min, and the *E. coli* O157:H7 was reduced by 2.03 log_10_CFU/g. For alfalfa seeds [41], after being treated with PAW for up to 3 h, only a decrease of 1.67 log_10_ CFU/g of *E. coli* O104 was observed. In addition, even after 1 h of PAW treatment, the KL value of *B. cereus* spores was not ideal, only 0.46 and 0.33 log_10_ CFU/mL [31,40]. Therefore, in a short sterilization time, the sterilization efficiency of PAW is significantly lower than that of AEW, especially for *E. coli*. However, the sterilization efficiency of AEW to *B. subtilis* is not ideal. It is necessary to increase the RCS content in AEW to improve the bactericidal efficiency against *B. subtilis*.

**Figure 2:**
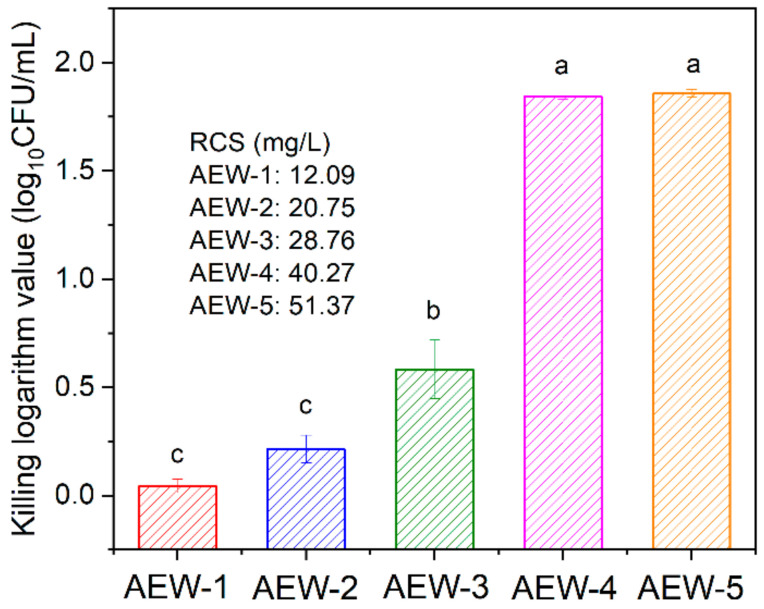
Sterilization effects of AEW with different RCS contents on the bacterial suspension of *B. subtilis*. Values with different lowercase letters above graph bars represent a significant difference (*p* < 0.05).

In previous studies, Han et al. [17] investigated the germicidal effects of AEW with different RCS on *E. coli*, *Salmonella* and *Yersinia*. As the RCS increased from 4.44 to 74.0 mg/L, the KL values of *E. coli*, *Salmonella* and *Yersinia* increased from 2.55, 2.84 and 0.46 log_10_CFU/mL to 8.46, 8.41 and 8.49 log_10_CFU/mL, respectively. Zhang et al. [6] studied the germicidal efficacy of AEW with RCS of 20, 60 and 100 mg/L on *B. cereus* spores, and their KL values were 0.22, 2,39 and 2.93, respectively. Therefore, the increase in RCS is beneficial to improve the sterilization efficiency. In this study, the sterilization efficiency of AEW against *B. subtilis* was investigated in the range of RCS content from 10 to 50 mg/L in Figure 2. When the RCS content was less than 20.75 mg/L, the sterilization efficiency increased with the increase in RCS content, and the KL value was between 0.04 and 0.21 log_10_CFU/mL. However, these sterilization efficiencies are not ideal. When the RCS content reaches 40.27 mg/L, the KL value can reach 1.84 log_10_CFU/mL. The AEW treatment with RCS between 20.75 and 40.27 mg/L was significantly different (*p* < 0.01). On this basis, even if the RCS content was further increased, the sterilization efficiency would not be further increased. This may be because the sterilization time of AEW is too short, and the sterilization time of 10 s is rarely used in previous studies. Al-Qadiri et al. [21] treated *B. cereus* with AEW, and its RCS was 60 mg/L and sterilization time was 5 min. Zhang et al. [19] treated *B. subtilis* spores with AEW, and its RCS was 120 mg/L and sterilization time was 2 min. Therefore, the sterilization efficiency of AEW with low RCS to *B. subtilis* is expected to be close to that for *E. coli*, and other methods should be considered. In addition, there is a notable phenomenon in Figure 1: the sterilization effect of PAW on *B. subtilis* is slightly better than that of *E. coli* (significant difference, *p* < 0.01). It can be assumed that if AEW is activated by plasma, the sterilization effect on *B. subtilis* can be improved. Therefore, the preparation and germicidal efficacy of plasma-activated AEW (PA-AEW) are investigated for the first time in this paper.

**Figure 3:**
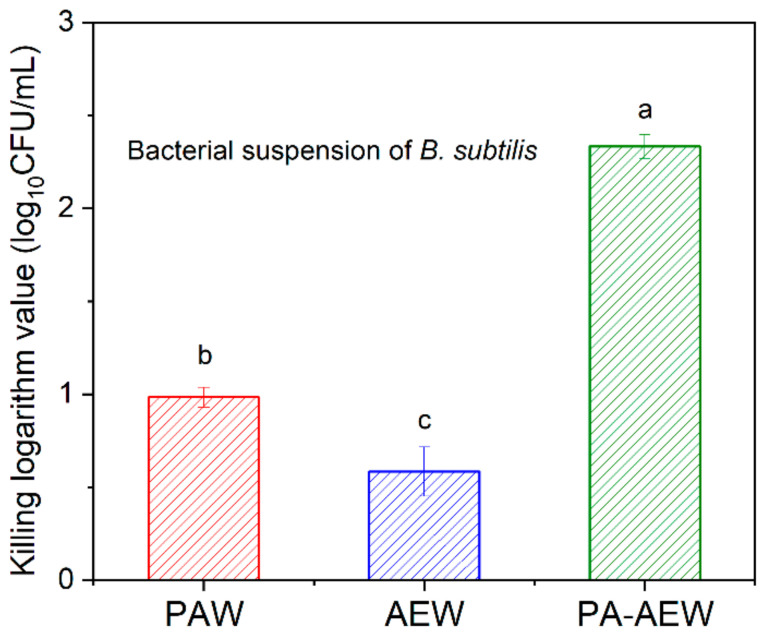
Sterilization effects of PAW, AEW and PA-AEW on the bacterial suspension of *B. subtilis*. Values with different lowercase letters above graph bars represent a significant difference (*p* < 0.05).

The sterilization effect of PA-AEW on *B. subtilis* is shown in Figure 3. After using plasma to activate AEW, its sterilization efficiency was significantly improved. The KL value of PA-AEW (RCS content = 28.76 mg/L) could reach 2.33 log_10_CFU/mL, which was significantly higher than that of AEW (the KL value = 0.58 log_10_CFU/mL). Additionally, it was higher than that of PAW (the KL value = 0.98 log_10_CFU/mL) with the same activation time (t = 3 min). The observed differences between PAW, AEW and PA-AEW were statistically significant (*p* < 0.01). Furthermore, compared with the AEW with the high RCS content (40.27 and 51.37 mg/L) in Figure 2, its KL value was also increased by 0.5 log_10_CFU/mL. The above analysis demonstrates that PA-AEW indeed has higher sterilization efficiency than AEW and PAW alone. This may be the synergistic effect between RCS in AEW and RONS produced by plasma activation. In order to further improve the sterilization efficiency of PA-AEW against *B. subtilis*, it is necessary to investigate the plasma activation of AEW with different RCS contents.

**Figure 4:**
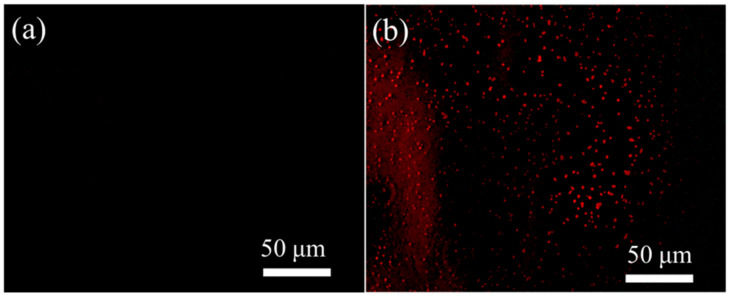
Fluorescence microscopy images of *B. subtilis* before (**a**) and after PA-AEW treatment (**b**).

To further verify the inactivation of *B. subtilis* exposed by PA-AEW, the cell viability was evaluated with a fluorescence probe (propidium iodide, PI) through fluorescence microscopy. The red fluorescing PI can only enter cells with injured cytoplasmic membranes, where it binds to nucleic acids and produces red fluorescence. After the PI staining for the control group, it was observed that *B. subtilis* cells were not stained (stimulated by green fluorescence) (Figure 4a), indicating that the vast majority of *B. subtilis* were alive. After the sterilization treatment with PA-AEW, the dense bacteria were seen in the field of vision to be stained red (Figure 4b), indicating that *B. subtilis* almost died.

**Figure 5:**
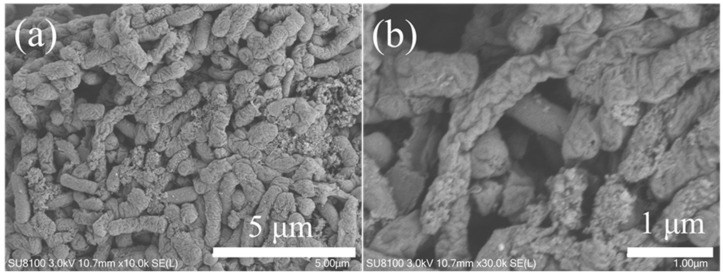
Scanning electron microscope (SEM) images of *B. subtilis* after PA-AEW treatment. (**a**) Scale bar: 5 μm, (**b**) scale bar: 1 μm.

Scanning electron microscopy (SEM) was used to verify the bactericidal effect of PA-AEW. Typical SEM images of *B. subtilis* under different magnifications after PA-AEW treatment are shown in Figure 5. It can be clearly seen that the outer surface of bacterial cells began to deform, filament and rupture. However, before the treatment, the surface of *B. subtilis* was rod-shaped with an intact membrane and smooth surface. This indicates that PA-AEW treatment is beneficial to destroy the integrity of bacterial cell membranes.

**Figure 6:**
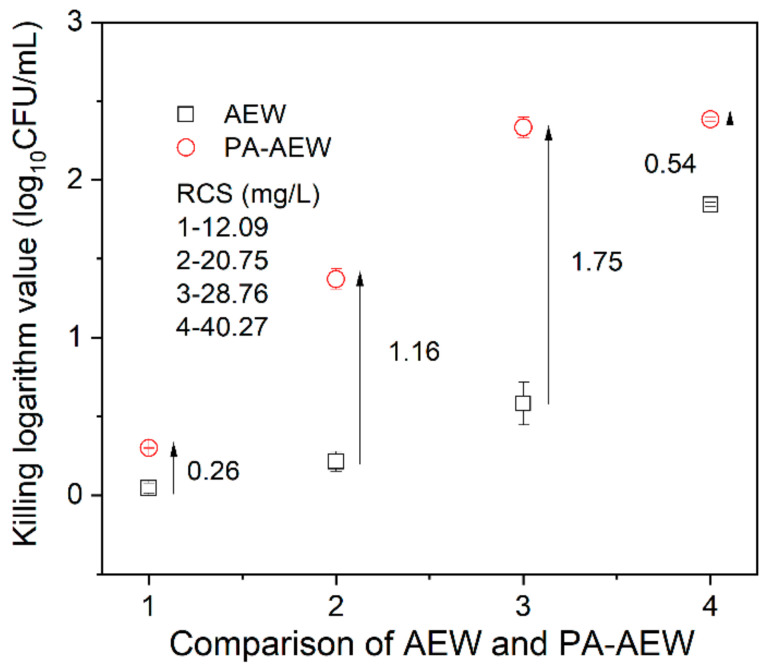
Comparison of sterilization efficiency of AEW and PA-AEW with different RCS on the bacterial suspension of *B. subtilis*.

**Figure 7:**
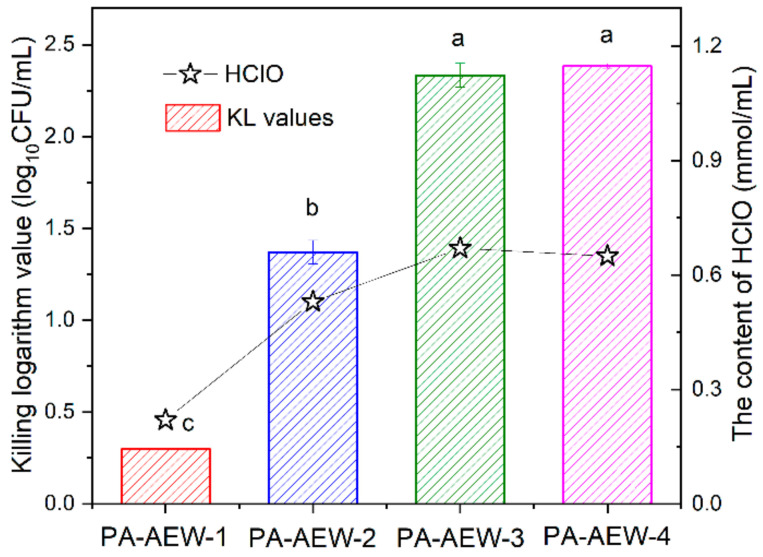
The relationship between the HClO content and sterilization efficiency of PA-AEW on the bacterial suspension of *B. subtilis*. Values with different lowercase letters above graph bars represent a significant difference (*p* < 0.05).

To further verify the sterilization efficiency of PA-AEW, the increment in the KL value of AEW with different RCS content after plasma activation was investigated, as shown in Figure 6. For AEW with RCS content of 12.09 mg/L, the KL value of PA-AEW was 0.3 log_10_CFU/mL, which only increased by 0.26 log_10_CFU/mL. For AEW with RCS content of 20.75 mg/L, the KL value of PA-AEW was 1.16 log_10_CFU/mL higher than that of AEW, and its increment was prominent. For AEW with RCS content of 28.76 mg/L, the increment in the KL value of PA-AEW was the largest, reaching 1.75 log_10_CFU/mL. However, for AEW with RCS content of 40.27 mg/L, the increment in the KL value of PA-AEW decreased to 0.54 log_10_CFU/mL. The above results indicate that the sterilization efficiency of AEW with different RCS contents was improved after plasma activation. This proves once again that there is synergy between RCS and RONS. The increasing ranges of sterilization efficiency were different, which may be related to the HClO content of RCS in AEW. HClO is the most active chlorine, which can effectively destroy and penetrate cell membranes, react with DNA and mitochondria and lead to microbial death. At the same time, HClO can also produce hydroxyl radical, which plays an antibacterial role through oxidation [7,9,10]. Therefore, the increase in HClO content will inevitably induce considerable synergy effects. The HClO content was directly proportional to the PA-AEW-1 (2, 3) sterilization efficiency in Figure 7 and there were significant differences between PA-AEW-1 (2, 3) (*p* < 0.01). For the PA-AEW-3 and PA-AEW-4, their HClO contents were close, so their sterilization efficiencies were not different (*p* > 0.05). The HClO content of PA-AEW-4 was close to that of PA-AEW-3, which was due to the partial decomposition of HClO during plasma activation. Besides RCS, there were RONS in PA-AEW. The influence of RONS on the sterilization efficiency of PA-AEW was illustrated later in this paper.

**Figure 8:**
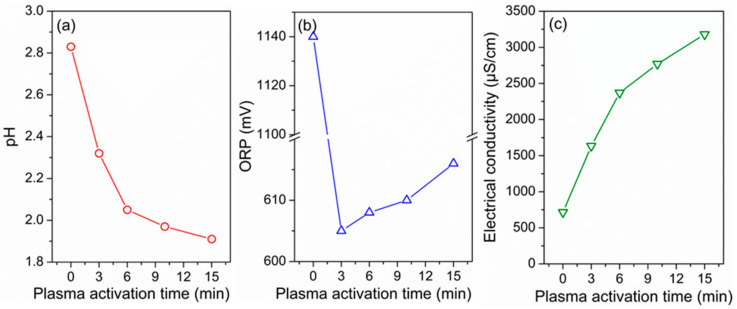
The values of pH (**a**), ORP (**b**) and EC (**c**) in PA-AEW accompanied by plasma activation time.

In order to explain the sterilization mechanism of PA-AEW, the physicochemical parameters of PA-AEW at different plasma activation times are analyzed in Figure 8. As for the pH value, with the extension of plasma activation time, the pH value gradually decreased. This was related to the generation of NO_3_^−^ and NO_2_^−^. The downward trend of pH was also pointed out in previous reports. Wang et al. [42] indicated that the pH of PAW decreased from 6.57 to 2.11 with the increase in activation time. Lin et al. [36] also proved that the pH decreased from 7.91 to 3.25 after the activation time of 90 s. In our studies, even though the initial pH of AEW was 2.83, it was further reduced to 1.91 after plasma activation. For the ORP value, the ORP value increased from 210 to 501 mV after the activation time of 90 s in Lin’s study [36]. In Bai’s study [40], the ORP value was increased to 550~573 mV after the activation time of 30~150 s. In our studies, the ORP value of AEW without plasma activation was 1140 mV, which was the nature of AEW itself. However, once AEW was activated by plasma, its ORP value dropped to 605 mV. This is because plasma activation directly produces a large number of electrons with strong reducibility, which greatly reduces the ORP value of AEW. However, when the plasma activation time was extended from 3 to 15 min, the ORP value increased slightly, which may be due to the slight increase in oxidizing species generated by the activation. As for conductivity, its measurement can reflect the change in ion property and concentration during plasma activation [28]. In this study, the electrical conductivity increased with the increase in plasma activation time, which is in agreement with the previous reports [35,42]. This is because with the increase in plasma activation time, more active substances are produced, which leads to the continuous increase in the conductivity of the solution.

The reactive species in PAW include both RCS and RONS. The influence of RCS content is analyzed in Figure 7. Therefore, the RONS in PAW is further analyzed below. The RONS usually includes long-lived species (H_2_O_2_, NO_3_^−^ and NO_2_^−^) and short-lived species (•OH, NO•, O_2_^−^, OONO_2_^−^ and ONOO^−^). The half-lives of •OH, NO•, O_2_^−^, OONO_2_^−^ and ONOO^−^ were proved to be 1 ns, a few seconds, 1.5 s and less than 1 s, respectively [25,26,27]. Because the half-lives of short-lived species are too short, the changes in long-lived species in PA-AEW with different plasma activation time are investigated below. Equations (1)–(13) illustrate the generation process of the three long-lived reactive species [25,26,27,28,32,34].

e + H_2_O → •H + •OH + e (1)

•OH + •OH → H_2_O_2_ (2)

e + N_2_ → •N + •N + e (3)

e + O_2_ → O + •O + e (4)

N + O → NO (5)

NO + O → NO_2_ (6)

NO + O_3_ → NO_2_ + O_2_ (7)

NO_2_ + O_3_ ↔ NO_3_ + O_2_ (8)

NO + NO_3_ ↔ NO_2_ + NO_2_ (9)

NO_2_ + NO_2_ + H_2_O → NO_2_^−^ + NO_3_^−^ + 2H^+^ (10)

NO + NO_2_ + H_2_O → 2NO_2_^−^ + 2H^+^ (11)

NO_2_^−^ + 3H^+^ + H_2_O → NO_3_^−^ + 2NO_2_ + 2H_3_^+^O (12)

NO_2_^−^ + H_2_O_2_ + H^+^ → ONOOH + 2H_2_O (13)

**Figure 9:**
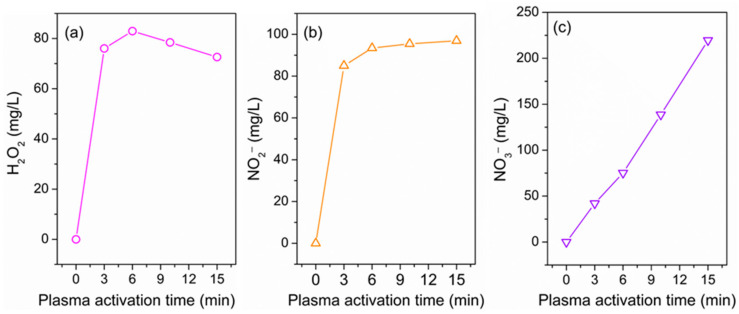
The contents of H_2_O_2_ (**a**), NO_2_^−^ (**b**) and NO_3_^−^ (**c**) in PA-AEW accompanied by plasma activation time.

For NO_3_^−^, its content increased linearly with the increase in plasma activation time in Figure 9c. When the plasma activation time was 3, 6, 10 and 15 min, the content of NO_3_^−^ was 42.02, 75.28, 138.7 and 219.6 mg/L, respectively. This is consistent with Ma’s findings [27], in which the NO_3_^−^ content increased by 1.954 mmol/L after 5 min activation. Meanwhile, in Wang et al.’s report [42], the concentration of NO_3_^−^ increased significantly to 1.0, 2.8 and 4.6 mmol/L after 2, 10 and 15 min, respectively. The increase in its content is mainly based on Equations (10) and (12). With the prolongation of plasma activation time, the above three reactions are continuously carried out, so the content of NO_3_^−^ is constantly increasing. The change trend of NO_2_^−^ content was different from that of NO_3_^−^ content in Figure 9. When the plasma activation time was less than 3 min, the NO_2_^−^ content sharply increased to 85 mg/L. After that, the content of NO_2_^−^ increased slowly. This change trend is related to Equations (10)–(13). When the reaction rates of the two types of reactions are close, the NO_2_^−^ content will be at a stable level. In Wang’s studies [42], the concentration of NO_2_^−^ reached the maximum value of 2.6 mmol/L after 5 min, and then slowly decreased. In Ma’s results [27], the NO_2_^−^ concentration also showed a trend of increasing at first and then decreasing slowly in the treatment process, and its maximum value was 0.109 mmol/L at 90 s. The change trend of H_2_O_2_ content was basically consistent with that of NO_2_^−^ content. The lack of a sustained increase in H_2_O_2_ content was due to its stability.

**Figure 10:**
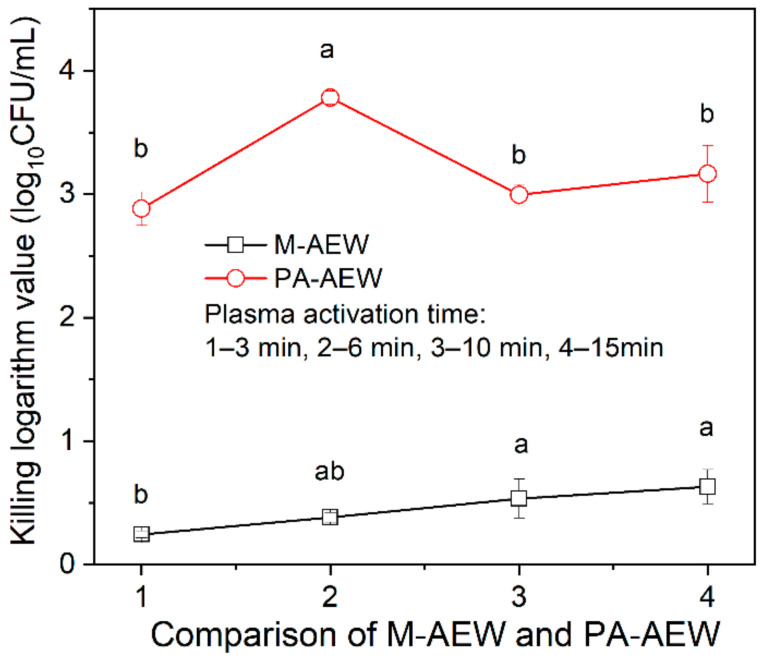
The comparison of sterilization efficiency on the bacterial suspension of *B. subtilis* for M-AEW and PA-AEW. Values with different lowercase letters above graph bars represent a significant difference (*p* < 0.05).

It is assumed that the long-lived species are important bactericidal factors in PA-AEW that can synergize with RCS, which can be verified by preparing modified AEW (M-AEW). According to the contents of H_2_O_2_, NO_3_^−^ and NO_2_^−^ in PA-AEW detected at different plasma activation times, different amounts of H_2_O_2_, NaNO_3_ and NaNO_2_ were added to AEW to ensure that the contents were close to those in PA-AEW. The sterilization effects of PA-AEW and M-AEW are compared in Figure 10. Even if the contents of three reactive species in M-AEW were close to those in PA-AEW, the sterilization efficiencies of all M-AEW were lower than those of PA-AEW. At the same time, Wang et al. [42] also pointed out that even if the pH value and NO_3_^−^ and NO_2_^−^ concentrations are the same, PAW is more effective than artificially acidified NO_x_^−^ solution. Compared with the sterilization efficiencies of AEWs in Figure 2, the sterilization efficiencies of M-AEW and AEW are close. Therefore, it is unrealistic to add H_2_O_2_, NO_3_^−^ and NO_2_^−^ reactive species to AEW to improve the sterilization efficiency. The high sterilization efficiency of PA-AEW may not come from the synergy between RCS and long-lived species in RONS. The synergistic bactericidal effect with RCS in PA-AEW should be the short-lived reactive RONS (•OH, NO•, O_2_^−^, OONO_2_^−^ and ONOO^−^)—this will be further analyzed and solved in follow-up research.

**Figure 11:**
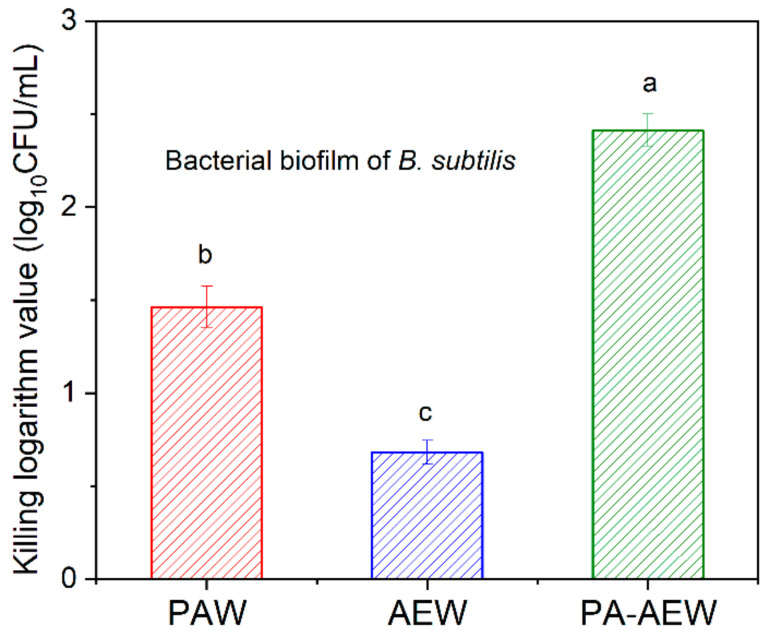
Sterilization effects of PAW, AEW and PA-AEW on the bacterial biofilm of *B. subtilis*. Values with different lowercase letters above graph bars represent a significant difference (*p* < 0.05).

Food-borne diseases are widespread in all parts of the world, and the hygiene problems caused by bacterial biofilm in food processing equipment are of particular concern, being directly related to human life safety and health. According to statistics [43,44,45,46,47,48], more than 80% of bacterial infections are related to bacterial biofilm in food processing equipment. Bacterial biofilm is a complex microbial community with multiple cells, with three-dimensional self-assembled extracellular polymeric substances (exopolysaccharide, protein, extracellular DNA, etc.). Compared with planktonic cells, biofilm cells are more resistant to fungicides, so they are extremely difficult to be eliminated. To further expand the application of PA-AEW, especially in the food industry, the bactericidal effect of PA-AEW on bacterial biofilm was investigated, shown in Figure 11. PA-AEW had an efficient bactericidal effect on the bacterial biofilm of *B. subtilis*. The KL value of PA-AEW was 2.41 log_10_CFU/mL, while that of PAW and AEW was only 1.46 and 0.68 log_10_CFU/mL, respectively. The observed differences between PAW, AEW and PA-AEW were statistically significant (*p* < 0.01). In Hussain’s previous research [22], when AEW was used alone to kill *B. cereus* biofilm, the RCS of AEW needed to reach 80 mg/L, and the treatment time was as long as 15 min. When PAW was used alone, Charoux et al. [35] indicated that the *E. coli* biofilm treated with PAW for 15 min did not show any significant decrease in count. Xu et al. [32] pointed out that it took 30 min of PAW treatment to reduce the *S. aureus* by 2.24 log_10_CFU/mL. However, the treatment time of this study was only 10 s, which shortened the sterilization time. The above results were as good as the above-mentioned sterilization effect on *B. subtilis* suspension, indicating that PA-AEW has high sterilization efficiency and important application prospects in food processing.

In recent years, PAW and AEW have been widely used for microbial safety and food quality. In this study, PA-AEW was prepared for the first time and had satisfactory sterilization efficiency, showing potential in food processing. However, there are still some problems to be considered and solved. First of all, what are the most effective and stable bactericidal factors in PA-AEW? What role do these short-lived sterilization factors play in the sterilization process? Secondly, how do RCS and RONS produce synergistic effects? Are there any new bactericidal factors? Finally, the preparation process of PA-AEW needs to be further optimized, such as activation distance, activation atmosphere and activation mode.

## 4. Conclusions

In this paper, AEW was activated by plasma for the first time, and a new food bactericide with high sterilization efficiency, namely PA-AEW, was obtained. When the sterilization time is only 10 s, the PA-AEW can achieve satisfactory bactericidal efficiency against *B. subtilis* (KL = 2.33 log_10_CFU/mL), significantly higher than that of AEW and PAW. Moreover, PA-AEW had an effective bactericidal effect on *B. subtilis* biofilm (KL = 2.41 log_10_CFU/mL), which was also superior to that of PAW and AEW. In the process of PA-AEW sterilization, through the analysis of sterilization factors and physicochemical parameters, there was a considerable synergistic effect between RCS and RONS. Furthermore, the comparison of the sterilization effect between the PA-AEW and M-AEW indicated that the high sterilization efficiency of PA-AEW may not be entirely due to the synergy between RCS and long-lived species in RONS. In future research, more attention should be paid to the synergistic bactericidal effect between RCS and short-lived reactive RONS (•OH, NO•, O_2_^−^, OONO_2_^−^ and ONOO^−^), although they are not easily detected separately. PA-AEW has high sterilization efficiency and rapidness, and it has wide application prospects in food preservation, food processing and food disinfection.

## Data Availability

Data sharing is not applicable to this article.

## Abbreviations

PA-AEW	plasma-activated acidic electrolyzed water
AEW	acidic electrolyzed water
PAW	plasma-activated water
M-AEW	modified AEW
RCS	reactive chlorine species
RONS	reactive oxygen and nitrogen species
KL	killing logarithm
ACC	available chlorine concentration
ORP	oxidation-reduction potential
EC	electrical conductivity
TMB	3,3′,5,5′-tetramethylbenzidine
NB	nutrient broth
TSA	tryptone soya agar
SEM	scanning electron microscopy
FL	Fluorescence microscopy
